# Awareness of cardiovascular disease risk and care received among Australian women with a history of hypertensive disorders of pregnancy: a cross-sectional survey

**DOI:** 10.1186/s12884-024-07018-5

**Published:** 2025-01-08

**Authors:** Kaylee Slater, Rachael Taylor, Clare E. Collins, Melinda Hutchesson

**Affiliations:** 1 School of Health Sciences, College of Health, Medicine and Wellbeing, University of Newcastle, Callaghan, New South Wales, NSW 2308 Australia; 2 Food and Nutrition Research Program, Hunter Medical Research Institute, Lot 1, Kookaburra Circuit, New Lambton Heights, NSW 2305 Australia; 3 School of Health Sciences, University of Sydney, Camperdown, New South Wales 2050 Australia

**Keywords:** Preeclampsia, Gestational hypertension, Primary care, General practice, Socioeconomic status, Cardiovascular disease, Cardiovascular risk, Preventative health, Prevention

## Abstract

**Background:**

Women with a history of hypertensive disorders of pregnancy (HDP), including chronic hypertension, gestational hypertension, and preeclampsia have an increased risk of cardiovascular disease (CVD). Current research suggests that general practitioners are unaware of women’s HDP history, and although ideally placed to follow-up with these women, there is limited understanding of current CVD prevention practices in women after HDP. Additionally, preeclampsia confers a higher CVD risk compared to other types of HDP, and Australian research suggests that lower socioeconomic status (SES) is associated with a higher incidence of both HDP and CVD. Therefore, the aim of the analysis was to investigate awareness of CVD risk and care received from health professionals among women with a history of HDP and examine differences between type of HDP and SES.

**Methods:**

Analysis of a cross-sectional survey of 293 Australian women with a history of HDP (from 2017 onwards). Data were analysed using basic descriptive statistics. To assess differences in HDP type and SES, one-way ANOVA was used to assess continuous variables and χ2 tests for categorical variables, with *P* < 0.05 considered statistically significant.

**Results:**

Most women with a history of HDP were unaware of their increased CVD risk (68%). Women with a history of preeclampsia, gestational hypertension or preeclampsia were more aware of CVD risk compared to those with chronic hypertension (*p* = 0.02). Regardless of HDP type or SES, women post-HDP were less likely to receive assessment and management of lifestyle CVD risk factors compared to blood pressure. Most women felt supported in managing stress and mental health, but not for managing body weight, smoking and sleep.

**Conclusions:**

Women with a history of HDP are unaware of their increased CVD risk and are not receiving recommended CVD preventative care, irrespective of HDP type and/or SES. Findings should be used to inform development of tailored CVD prevention interventions in the primary care setting for women following HDP.

**Supplementary Information:**

The online version contains supplementary material available at 10.1186/s12884-024-07018-5.

## Introduction

Hypertensive disorders of pregnancy (HDP), which include chronic hypertension, gestational hypertension, and preeclampsia affect approximately 8–10% of pregnancies worldwide [1]. Chronic hypertension is pre-existing hypertension or diagnosis of hypertension before 20 weeks gestation and gestational hypertension is the new onset of hypertension, diagnosed after 20 weeks gestation [2]. Preeclampsia is a multi-system disorder, that includes hypertension plus involvement of another organ system, most commonly the kidneys where proteinuria will be diagnosed [2]. In a cross-sectional study using linked population databases from Australia, out of 250,173 women 9.8% had HDP during pregnancy, with 4.2% having preeclampsia, and 4.3% gestational hypertension [3]. It is now well established that women diagnosed with HDP have a two-to-four-fold increased risk of developing cardiovascular disease (CVD), especially hypertension and stroke, within 10 years postpartum [4].

Recent research suggests that preeclampsia has a greater adverse impact on subsequent CVD risk than gestational hypertension [5]. However, there is also emerging research of the impact of gestational hypertension. For example, in a Danish cohort of 1.5 million primiparous women followed up post-pregnancy (1–20 years), women with gestational hypertension had a higher risk of developing chronic hypertension, while preeclampsia seemed to confer the highest risk for future CVD [6]. While the American Heart Association recognises preeclampsia and gestational hypertension as female risk factors for CVD and stroke, it does not mention chronic hypertension in pregnancy [5, 7, 8]. Additionally, there is currently a lack of research regarding chronic hypertension during pregnancy and CVD risk post-pregnancy.

Previous research suggests lower socioeconomic status (SES) is associated with a higher incidence of both HDP and CVD [9, 10]. In an analysis of epidemiological characteristics of HDP from a cohort study, Wang et al. found that both incidence and prevalence of HDP were highest in countries with lower sociodemographic indices, for example all countries in the African continent [9]. Additionally, in Australia, the incidence of HDP decreased as sociodemographic index increased [9]. Interestingly, a 2018 database cohort study undertaken in Korea suggested that even with universal healthcare, women with lower SES had higher rates of preeclampsia [11]. Kim et al. suggested that individuals with a lower SES were also less likely to utilise prenatal care, and therefore were at higher risk of pregnancy complications, such as HDP [11].

Despite a large body of research highlighting the relationship between HDP and CVD, it appears that women with a history of HDP remain largely unaware of the increased risk [12]. A recent 2019 scoping review (*n* = 12 studies) examining women’s knowledge of CVD after HDP reported that women had either limited, or no knowledge of their increased CVD risk [12]. While this review reiterated that awareness of CVD risk was low, it also acknowledged that little evidence was collected addressing how to educate women on their CVD risk [12]. To date, there are limited clinical trials for CVD prevention in women after HDP [13–15]. A 2019 systematic review of randomised controlled trials focused on reducing CVD risk after HDP published between 2008 and 2019, identified just two RCTs that focused on reducing CVD risk after HDP [13]. Lui et al. also identified four additional RCTs that were not yet completed at the time of publication in 2019, however since then, just two of these studies have published results [16, 17]. Consequently, understanding the care women with a history of HDP are currently receiving and whether this differs depending on the type of HDP and SES, can be used to inform clinical guidelines and practice.

Therefore, the primary aim of the current study was to investigate whether women after HDP were aware of their CVD risk and understand CVD care they received from health professionals. The secondary aim was to evaluate whether care differed by HDP type and SES, within the primary health care setting, as general practitioners (GPs) are ideally placed to provide CVD risk assessment and management for these women postpartum.

## Methods

### Study design

The current study is an analysis of a cross-sectional survey. The purpose of this analysis was to understand CVD risk awareness and current CVD prevention practices for women after HDP. A secondary aim was to determine whether CVD prevention practices differed between women with HDP subtypes (chronic hypertension, gestational hypertension and preeclampsia) and by SES. Results from the survey on perceived barriers and enablers, as well as potential strategies for obtaining advice after HDP about CVD from health professionals have been previously reported [18]. Checklist for Reporting Results of Internet E-Surveys (CHERRIES) was used to report results from the survey [19] and Strengthening the Reporting of Observational Studies in Epidemiology (STROBE) statement was applied for the reporting of this manuscript [20]. Ethics approval was received from the University of Newcastle Human Research Ethics Committee (H-2021-0415).

### Participants

Women were eligible if they were over 18 years old, currently living in Australia and had self-reported chronic hypertension, gestational hypertension or preeclampsia in one or more pregnancies from 2017 until August 2022. There were no other exclusion criteria.

### Recruitment

Australian women were recruited online via social media, such as Facebook and Instagram. Researchers disseminated promotional material to respective HDP-focused Facebook groups, including Preeclampsia, Eclampsia and HELLP Syndrome Survivors Global Support Network and the Centre of Perinatal Excellence, as well as other Facebook groups targeted to Australian mothers. Researchers also liaised with The Australian Action on Preeclampsia [21], Australasian Birth Trauma Association [22] and Mama Tribe [23], who shared recruitment material on their social media accounts. The study was also promoted by Australian experts such as fertility and pregnancy dietitians, obstetricians, and fertility specialists, with recruitment material shared on their professional social media accounts including Instagram, Facebook, and Twitter. All women who returned a survey were given the opportunity to go into a prize draw to win one of 10 e-gift cards valued at AUD$200 as an incentive to participate.

### Data collection

All data were collected and managed using Research Electronic Data Capture (REDCap) tools hosted at the Hunter Medical Research Institute, Australia. REDCap (version 12.5.5 by Vanderbilt University) is a secure, web-based software platform designed to support data capture for research studies [24, 25].

### Participant survey

The survey (Additional Files) contained 30 closed-ended questions and one open-ended question. It captured key sociodemographic characteristics of women following HDP (e.g., age, education level, postcode), the extent of CVD prevention services provided by their respective health professionals since their HDP diagnosis and perceived barriers and enablers, as well as potential strategies to obtaining advice after HDP about CVD from healthcare professionals. The current analysis reported on 16 questions including sociodemographic characteristics of women following HDP, awareness of CVD risk and the extent of CVD prevention services provided to women, and explored differences by HDP type and SES.

### Awareness of CVD risk

Participants were asked the question “Before reading the information about this survey, were you aware that after experiencing a high blood pressure problem during pregnancy, a woman has a greater risk of developing heart disease?” to determine their awareness of CVD risk. If they answered yes to this question, they were then asked another closed-ended question about how they became aware of this risk, with options such as ‘told by a health professional’.

### Current practice

Participants were asked to self-report the type of health professional involved in providing their CVD preventative care after HDP for CVD risk markers (obstetric history, blood pressure, blood lipids and blood glucose levels) and lifestyle CVD risk factors (diet, physical activity, smoking status, alcohol consumption, sleep habits, body weight and stress/mental health). They were also asked whether they felt supported by their GP to make changes to lifestyle CVD risk factors using a Likert scale from one to five, where 1 = Not at all supported and 5 = Very supported.

### Type of HDP

Participants were provided with definitions of the three subtypes of HDP and were asked “which type of blood pressure problem were you diagnosed with in your most recent pregnancy?” As participants may be diagnosed with more than one subtype of HDP during a single pregnancy, such as gestational hypertension and preeclampsia, they were allowed to choose more than one option. Self-reporting of perinatal outcomes is shown to be reliable, specifically gestational hypertension in New South Wales (NSW) [26] and preeclampsia [27], and participants were provided with definitions of each subtype of HDP at the start of the survey. Participants were therefore categorised as either chronic hypertension, gestational hypertension or preeclampsia.

### Socioeconomic status

Socioeconomic status was classified using the Socio-Economic Indexes for Areas (SEIFA) indexes [28]. The SEIFA indexes rank areas in Australia according to their relative socioeconomic advantage and disadvantage which is based on key dimensions including income, education, employment, occupation, and housing recorded in the Census data [28]. Postcodes were matched using the most current Postal Area Index of Relative Socioeconomic Advantage and Disadvantage 2021. Quintiles were used where quintile 1 represents the lowest SES group (i.e., most disadvantage), whereas quintile 5 represents the highest (i.e., highest advantage).

### Statistical analysis

All statistical analyses were performed using the Stata 16.1 (StataCorp LLC, College Station, Texas USA) [29]. For this study participants were classified into either chronic hypertension (*n* = 17), gestational hypertension (*n* = 104) or preeclampsia (*n* = 173). Participants who selected both chronic hypertension and preeclampsia (*n* = 10) and both gestational hypertension and preeclampsia (*n* = 26) were placed in the preeclampsia group given that current literature suggests a higher risk of future CVD with preeclampsia [4]. Participants who selected chronic hypertension and gestational hypertension were excluded from the current analysis as an individual cannot be diagnosed with both health conditions (*n* = 9). Continuous data were assessed for normality using the Shapiro-Wilks test of normality. The primary aim was to investigate awareness of CVD risk and the care received from health professionals among women with a history of HDP, which was analysed via basic descriptive statistics. The secondary aim of the analysis was to examine the association between type of HDP and SES and CVD risk awareness and care received from health professionals after HDP. Analysis of variance (one-way ANOVA) tests were used to assess continuous variables and χ2 tests for categorical variables for significant differences. *P*-values of < 0.05 were considered statistically significant.

## Results

### Participants

**Fig. 1:**
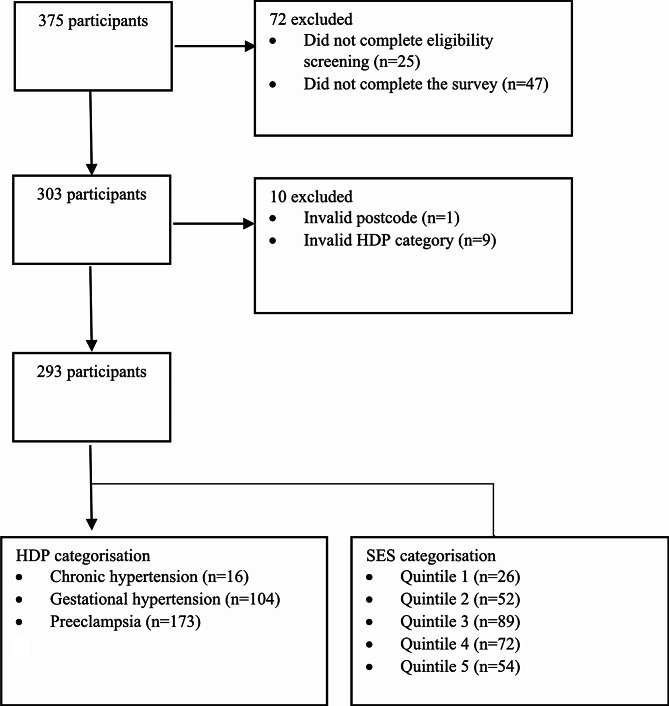
Participant flow chart

Figure 1 presents the flow of the 293 participants into the study, categorised by HDP type and SES quintiles.

### Demographic characteristics

Table 1 summarises the demographic characteristics of participants. The majority of participants had been diagnosed with HDP since 2020 (*n* = 201, 69%). Participants were predominantly higher income earners (*n* = 185, 63%), tertiary educated (194, 66%) and living in Victoria, or NSW (215, 73%), Australia. Obstetricians were the most common health professional involved in women’s care (240, 82%), whereas majority did not have a GP involved in their care throughout pregnancy (*n* = 204, 69%). There were statistically significant differences in country of birth among women with different HDP types (*p* = 0.04) (Additional Files, Table 1). There were statistically significant differences in the state of residence among women by SES (*p* < 0.00), where more women living in NSW had postcodes in quartile 2 (*n* = 40, 71%) and quartile 3 (58, 64%) than other states (Additional Files 1, Table 2).

**Table 1:** Demographic characteristics of Australian women with a history of HDP

Variables	Value	
Age (years)	Mean	(SD)
	33.40	4.81
**Demographic characteristics (*n*** **=** **293)**	**Total n**	**Percentage (%)**
**Marital status**		
Single	6	1
De facto	59	20
Married	225	77
Divorced/separated	3	1
**Country/area of birth**		
Australia/New Zealand	271	92
North America (USA and Canada)	2	1
Central/South America	3	1
United Kingdom/Ireland	8	3
Asia	7	2
Africa/South Africa	2	1
**Australian state of residence**		
New South Wales	146	50
Victoria	69	24
Queensland	37	13
South Australia	9	2
Australian Capital Territory	10	2
Northern Territory	3	1
Western Australia	16	5
Tasmania	3	1
**Highest level of education**		
No formal qualifications	1	1
School Certificate (Year 10 or equivalent)	9	3
High school Certificate (Year 12 or equivalent)	20	7
Trade/Apprenticeship	7	2
Certificate/Diploma	62	21
University undergraduate degree	124	42
Higher university degree (postgraduate)	70	24
**Household income (AUD$)**		
$3,000 + per week or $156,000 per year or more	59	20
$2,000 - $2,999 per week or $104,000 $155,999 per year	50	17
$1,750 - $1,999 per week $91,000 - $103,999 per year	76	26
$1,500 - $1,749 per week $78,000 - $90,999 per year	38	13
$1,250 - $1,499 per week $65,000 - $77,999 per year	21	7
$1,000 - $1,249 per week $52,000 - $64,999 per year	20	7
$800 - $999 per week $41,600 - $51,999 per year	13	4
$650 - $799 per week $33,800 - $41,599 per year or less	9	3
Don’t want to answer	7	2
**Most recent diagnosis of HDP**		
2017	22	6
2018	30	10
2019	40	14
2020	71	24
2021 onwards	130	44
**Type of health professional involved in care during pregnancy**		
General practitioner	89	30
**Obstetrician** Public Private	117 123	40 42
**Midwife** Public Private	134 21	54 7

AUD, Australian Dollars; HDP, hypertensive disorder of pregnancy; USA, United States of America

### Current CVD prevention practice after HDP

Women’s awareness of increased CVD risk after HDP is reported in Table 2. Many women in the current sample were unaware of their increased CVD risk (*n* = 199, 68%). Of the 94 who were aware, they were most likely to have been informed by their obstetrician (*n* = 30, 32%) or through their own research (*n* = 34, 36%). There was a statistically significant difference in CVD risk awareness among HDP subtypes (*p* = 0.01). Women with a history of preeclampsia (39%) were the most likely to be aware (Additional Files 1, Table 3). Women with a history of gestational hypertension were the least likely to be aware of their increased CVD risks (*n* = 22, 21%). There were no differences in CVD risk awareness by SES.

Women’s CVD assessment by health professionals is reported in Table 2, and findings by type of HDP/SES in Additional Files, Tables 3 and 4. This table demonstrates the number of women who had at least one health professional assess these CVD risk markers. In the current sample, the majority of participants had their blood pressure (81%) and obstetric history (72%) assessed, although most were reportedly not asked about lipid (29%) or blood glucose levels (39%). There were statistically significant differences in the number of women by HDP-subtype who were asked about their obstetric history (*p* = 0.01). Sixty-six percent of women with a history of preeclampsia had been asked about their obstetric history, whereas over 80% with either chronic hypertension or gestational hypertension had been asked about their obstetric history.

Many women in this sample had their stress/mental health assessed (76%), followed by physical activity (*n* = 59%) and diet (51%), whereas less than 50% of women were assessed for other lifestyle CVD risk factors. There were no statistically significant differences in the number of women whose lifestyle CVD risk factors had been assessed by HDP or SES subtype (*p* > 0.05).

**Table 2:** Awareness of CVD risks, CVD risk marker and lifestyle CVD risk factor assessment by a health professional, self-reported by women with a history of HDP

	Total *n*	Percentage (%)	HDP Type *p*-value	SES *p*-value
**Awareness of CVD risk (*n*** **=** **293)**	94	32	0.01	0.75
**CVD risk markers assessed by a health professional (*n*** **=** **293)**				
Obstetric history	212	72	0.01	0.23
Blood pressure	236	81	0.13	0.82
Blood lipids	84	29	0.65	0.87
Blood glucose/insulin	113	39	0.20	0.64
**Lifestyle CVD risk factors assessed by a health professional (*n*** **=** **293)**				
Diet	148	51	0.28	0.25
Physical activity	172	59	0.05	0.62
Smoking status	136	46	0.59	0.60
Alcohol consumption	134	46	0.75	0.43
Sleep habits	132	45	0.99	0.77
Body weight/ overweight and obesity	111	38	0.50	0.50
Stress/mental health	223	76	0.84	0.41

CVD: cardiovascular disease. HDP: hypertensive disorders of pregnancy. SES: socioeconomic status

Table 3 summarises the level of perceived support women received from GPs in regard to lifestyle-related CVD risk factors. Participants could select “Not applicable to me”, which was selected by 10% (*n* = 30) of participants for diet, 11% (*n* = 31) for physical activity, 65% (*n* = 189) for smoking, 53% (*n* = 155) for alcohol intake, 16% (*n* = 48) for sleep, 16% (*n* = 48) for weight management and 7% (*n* = 20) for mental health support. Over 60% of women with a history of HDP (*n* = 188) felt supported (either very or somewhat supported) by their GP when it came to their mental health. Of those who felt getting support for smoking and alcohol consumption was relevant to them, < 30% felt supported by their GP to manage smoking (*n* = 46) or alcohol consumption (*n* = 72). Less than 50% of women with a history of HDP felt supported by their GP when it came to managing their diet (*n* = 138), physical activity levels (*n* = 133), or body weight (*n* = 124). There were no statistically significant differences between the subtypes of HDP or SES and perceived level of support by their GPs (*p* > 0.05).

**Table 3:** Level of support women perceived from general practitioners to make changes to lifestyle CVD risk factors, as self-reported by women with HDP history

Lifestyle CVD risk factor (*n* = 293)	Total *n*	Percentage (%)	HDP Type *p*-value	SES *p*-value
**Diet**				
Very supported	72	25	0.41	0.20
Somewhat supported	66	23		
Neutral	77	26		
Not very supported	34	12		
Not at all supported	14	5		
**Physical activity**				
Very supported	64	22	0.50	0.40
Somewhat supported	69	24		
Neutral	77	26		
Not very supported	40	14		
Not at all supported	12	4		
**Smoking status**				
Very supported	35	12	0.18	0.42
Somewhat supported	21	7		
Neutral	31	11		
Not very supported	8	3		
Not at all supported	9	3		
**Alcohol consumption**				
Very supported	43	15	0.65	0.49
Somewhat supported	29	10		
Neutral	43	15		
Not very supported	14	5		
Not at all supported	9	3		
**Sleep habits**				
Very supported	53	18	0.30	0.84
Somewhat supported	57	19		
Neutral	71	24		
Not very supported	46	16		
Not at all supported	18	6		
**Body weight/overweight and obesity**				
Very supported	68	23	0.56	0.15
Somewhat supported	56	19		
Neutral	74	25		
Not very supported	31	11		
Not at all supported	16	5		
**Stress/mental health**				
Very supported	110	38	0.21	0.35
Somewhat supported	78	27		
Neutral	50	17		
Not very supported	23	8		
Not at all supported	12	4		

CVD: cardiovascular disease. HDP: hypertensive disorders of pregnancy. SES: socioeconomic status

## Discussion

The current survey of 293 Australian women with a history of HDP examined their awareness of future CVD risk and the care received from their health professionals, with results compared among HDP subtypes and by SES. Results suggest that regardless of HDP-type or SES, women with a history of HDP are unaware of their increased CVD risk. Additionally, when receiving care from health professionals, they are less likely to receive assessment and management for lifestyle-related CVD risk factors, including diet and physical activity, compared to blood pressure or obstetric history. Most women with HDP history in the current sample felt supported by their GP in regard to their mental health, but less supported to manage body weight, reduce smoking and improve sleep.

International Society for the Study of Hypertension in Pregnancy (ISSHP) guidelines suggest that following HDP, women should be advised of their increased risk of CVD and stroke [30]. In the current sample, less than 50% of women were aware of their increased CVD risk. A 2019 scoping review of 12 studies found that women’s knowledge of CVD risk after HDP differed according to the type and severity of HDP, where CVD risk awareness was higher in women who were diagnosed with chronic hypertension (75%) compared to those diagnosed with preeclampsia without eclampsia (43%) [12]. By contrast in the current study, 31% of women with chronic hypertension and 21% with gestational hypertension were aware of their increased CVD risk, compared to 39% of women diagnosed with preeclampsia. There are a variety of factors previously reported as contributing to women’s limited awareness of their CVD risk, including poor communication between health professionals of obstetric history, inconsistent standards for transition from hospital to community care and limited education and resources for health professionals caring for women after HDP [31]. In a recent survey of 266 Australian women, women with a history of HDP (*n* = 174) had high knowledge of risk of HDP reoccurrence in a future pregnancy, but only moderate knowledge of future CVD events [32]. Additionally, only 43% of women with a history of HDP suggested they discussed future risk of chronic hypertension, and when asked about education preferences after HDP, 80% preferred receiving information about long-term health from a healthcare provider or key organisation (60%), compared to social media (47%) [32]. Additionally, in a 2023 cross-sectional survey of 438 women diagnosed with gestational hypertension or preeclampsia, women who reported awareness of increased CVD risk were also more likely to have engaged in CVD risk assessment [33]. Atkinson et al. found that 55% of women who were aware of CVD risks had annual blood pressure checks, compared to 38% of those who were unaware (*p* < 0.01), with similar results seen for at least one assessment of blood cholesterol (*p* < 0.01) and blood glucose (*p* = 0.03) [33]. This suggests that despite type of HDP diagnosis, in order to improve CVD risk assessment and management post-HDP, a vital first step is improving women’s awareness of the relationship between CVD and HDP, and then to understand how they prefer to receive CVD care.

ISSHP guidelines and the Society of Obstetric Medicine Australia and New Zealand (SOMANZ) guidelines suggest that after HDP, women should be encouraged to see a GP for an annual blood pressure check, a five-yearly check of plasma lipids and blood glucose levels and to adopt a healthy lifestyle [30, 34]. Most women in the current study reported that their blood pressure was measured post-HDP, although a smaller proportion (< 40%) reportedly had their lipids and/or blood glucose levels measured. Similar results were observed in a 2018 survey of 127 Australian women with a recent history of preeclampsia (≤ 2 years), where Hutchesson et al. asked participants whether they had received advice or screening as per the SOMANZ guidelines [35]. Although 95% reportedly had their blood pressure measured, only 41% had cholesterol or glucose checked, and fewer received advice on lifestyle risk factors [35]. Women in the current study reported that health professionals were more likely to assess obstetric history and blood pressure compared to lifestyle risk factors, with the exception of mental health/stress which had been assessed for 76% of women. These results are similar to a survey of Australian GPs, where GPs were more likely to report assessing and managing traditional CVD risk factors than female-specific risk factors, such as HDP or lifestyle risk factors [18]. This may reflect a GP’s confidence in managing traditional CVD risk factors such as blood pressure, and less familiarity with CVD lifestyle prevention. This is evident in a 2018 systematic review of GPs perspectives on the prevention of CVD, where some GPs suggested that nutrition education was outside their area of expertise, and that other clinicians, such as dietitians, were better placed to provide lifestyle advice [36].

In the current study, type of HDP was only related to GPs assessment of CVD risk factors when it came to assessment of obstetric history. Women with a history of preeclampsia were less likely to be asked about their obstetric history (66%) than other subtypes of HDP (> 80%). However, the current study did not find any differences in CVD preventative care based on an indicator of SES. When accessing health care, Filc et al. indicated that those with lower SES were more likely to visit a GP instead of a specialist, present to the emergency department and be hospitalised for longer than those with higher SES [37]. In a retrospective analysis of CVD screening from a General Practice Database in Melbourne, Australia, among the 149,306 patients, females and those of lower SES were less commonly screened for CVD risk factors [38]. Additionally, previous research suggests that those of lower SES are more likely to develop pregnancy complications, such as HDP [39–41]. It is important that interventions targeted at CVD risk reduction in women following HDP must also target SES disparity. However, it must be noted that recruitment in the current study predominately occurred online and captured a higher SES sample from metropolitan NSW and Victoria in Australia. Additionally, the marker used for SES in the current study provides a broad measure of economic and social conditions of people and households within an area [28]. Therefore, the range of health disparities may not have been captured to be able to observe a meaningful difference.

Continuity of care after pregnancy is an important contributor to preventing and managing long-term health complications. In a recent qualitative interpretive study, Ray et al. describes the experiences of Australian women in receiving information about pregnancy complications from health professionals [42]. Ray et al. reports that women found communication from their health professionals distressing, and were concerned by the delays in education [42]. In contrast, while the current study investigated care after HDP, rather than during pregnancy, regardless of HDP type or SES, the majority of women felt supported when it came to managing stress and mental health. However, while a large percentage of women in the current sample were reportedly not asked about lifestyle CVD risk factors, many felt supported by their GP to make changes to their lifestyle. This reflects an important opportunity for GPs and the primary care setting in providing lifestyle CVD prevention to women after HDP. Gamble et al. also suggested that although this clinical area falls between GPs, obstetrics/gynaecology and cardiology, the GPs are best placed to follow-up with these women once they are discharged into the community [43].

As previously noted, ISSHP and SOMANZ guidelines recommend women with a history of HDP engage in CVD prevention, although there is little information on GPs role in this care [2, 44]. In a recent qualitative study of Australian mothers and GPs, the GPs voiced concerns about a lack of consistent guidelines for their involvement in routine postpartum care [45]. A 2019 review summarising guidelines for the prevention of CVD after HDP identified 16 guidelines documenting the follow-up of women with HDP, and eight providing recommendations beyond the immediate postpartum period [43]. While the majority of guidelines recommend that both women and their GP are informed of CVD risks after HDP, there was no consensus of how or when to initiate follow-up, or which risk factors should be included in screening [43]. Therefore, although ideally placed to provide postpartum CVD risk assessment and management to women after HDP, the lack of consistent guidelines for the role of GPs is likely influencing the provision of care to all women.

There are several limitations in the present study that should be noted when interpretating findings. The current sample was predominantly from two states in Australia (NSW and Victoria) and therefore is not representative of all Australian women. The majority of participants in this sample had experienced HDP within the past two-years. While this allows us to describe current practice, we cannot explore change in practice over time, nor the effect that COVID-19 may have had on care received. Lastly, the survey did not consider care received during pregnancy e.g., where women birthed and maternity models of care engaged with, or other pregnancy complications, such as gestational diabetes. Therefore, authors could not evaluate whether postpartum care differed with the different models of maternity care or the presence of additional or competing health conditions.

## Conclusions

The current study provides valuable information on awareness of CVD risk and current CVD preventive care provided to women with a history of HDP, specifically understanding differences between HDP type and SES. This analysis suggests that the majority of women with a history of HDP are currently unaware of their increased CVD risk and are not receiving CVD preventative care consistent with recommendations in international and national guidelines, irrespective of HDP type and/or SES. It is important that women and their primary care providers are aware of obstetric history, as well as potential long-term complications that may arise because of pregnancy complications such as HDP. Women frequently become disconnected from ongoing medical care after birth, and therefore need to be made aware that the postpartum period is a critical time to focus on long-term CVD risk reduction [46]. These findings can be used to guide development and implementation of tailored CVD prevention and intervention programs for women following HDP in the primary care setting.

## Electronic supplementary material

Below is the link to the electronic supplementary material.

Supplementary Material 1

## Data Availability

The original contributions presented in the study are included in the article or supplementary material, further inquiries can be directed to the corresponding author.

## Abbreviations

HDP: Hypertensive disorders of pregnancy
CVD: Cardiovascular disease
SES: Socioeconomic status
GPs: General practitioners
CHERRIES: Checklist for reporting results of internet e-surveys
SEIFA: Socio-economic indexes for areas
ANOVA: Analysis of variance
ISSHP: International society for the study of hypertension in pregnancy
SOMANZ: Society of obstetric medicine Australia and New Zealand

## References

[1] Ikem E, Halldorsson TI, Birgisdóttir BE, Rasmussen MA, Olsen SF, Maslova E. Dietary patterns and the risk of pregnancy-Associated Hypertension in the Danish National Birth Cohort: a prospective longitudinal study. BJOG Int J Obstet Gynaecol. 2019;126(5):663–73. 10.1111/1471-0528.15593 30675768

[2] Lowe SA, Bowyer L, Lust K, McMahon LP, Morton M, North RA, et al. Somanz guidelines for the management of Hypertensive disorders of pregnancy 2014. Aust New Z J Obstet Gynaecol. 2015;55(5):e1–29. 26412014 10.1111/ajo.12399

[3] Roberts CL, Algert CS, Morris JM, Ford JB, Henderson-Smart DJ. Hypertensive disorders in pregnancy: a Population-based study. Med J Australia. 2005;182(7):332–5. 15804223 10.5694/j.1326-5377.2005.tb06730.x

[4] Wu R, Wang T, Gu R, Xing D, Ye C, Chen Y, et al. Hypertensive disorders of pregnancy and risk of Cardiovascular Disease-related morbidity and mortality: a systematic review and Meta-analysis. Cardiology. 2020;145(10):633–47. 32841945 10.1159/000508036

[5] Poon LC, Nguyen-Hoang L, Smith GN, Bergman L, O’Brien P, Hod M, et al. Hypertensive disorders of pregnancy and long-term Cardiovascular Health: Figo Best Practice advice. Int J Gynecol Obstet. 2023;160(S1):22–34. 10.1002/ijgo.14540 36635079

[6] Behrens I, Basit S, Melbye M, Lykke JA, Wohlfahrt J, Bundgaard H, et al. Risk of Post-pregnancy Hypertension in Women with a history of Hypertensive disorders of pregnancy: Nationwide Cohort Study. BMJ. 2017;358:j3078. 28701333 10.1136/bmj.j3078 PMC5506851

[7] Bushnell C, McCullough LD, Awad IA, Chireau MV, Fedder WN, Furie KL, et al. Guidelines for the Prevention of Stroke in Women. Stroke. 2014;45(5):1545–88. 24503673 10.1161/01.str.0000442009.06663.48 PMC10152977

[8] Benschop L, Duvekot JJ, Roeters van Lennep JE. Future risk of Cardiovascular Disease Risk factors and events in women after a hypertensive disorder of pregnancy. Heart. 2019;105(16):1273. 31175138 10.1136/heartjnl-2018-313453 PMC6678044

[9] Wang W, Xie X, Yuan T, Wang Y, Zhao F, Zhou Z, et al. Epidemiological Trends of Maternal Hypertensive Disorders of Pregnancy at the Global, Regional, and national levels: a Population-based study. BMC Pregnancy Childbirth. 2021;21(1):364. 33964896 10.1186/s12884-021-03809-2 PMC8106862

[10] Schultz WM, Kelli HM, Lisko JC, Varghese T, Shen J, Sandesara P, et al. Socioeconomic Status and Cardiovascular outcomes. Circulation. 2018;137(20):2166–78. 29760227 10.1161/CIRCULATIONAHA.117.029652 PMC5958918

[11] Kim MK, Lee SM, Bae S-H, Kim HJ, Lim NG, Yoon S-J, et al. Socioeconomic status can affect pregnancy outcomes and complications, even with a Universal Healthcare System. Int J Equity Health. 2018;17(1):2. 29304810 10.1186/s12939-017-0715-7 PMC5756361

[12] Roth H, LeMarquand G, Henry A, Homer C. Assessing knowledge gaps of women and Healthcare Providers concerning Cardiovascular Risk after Hypertensive disorders of Pregnancy-a scoping review. Front Cardiovasc Med. 2019;6:178. 31850374 10.3389/fcvm.2019.00178 PMC6895842

[13] Lui NA, Jeyaram G, Henry A. Postpartum interventions to Reduce Long-Term Cardiovascular Disease Risk in women after Hypertensive disorders of pregnancy: a systematic review. Front Cardiovasc Med. 2019;6:160. 31803757 10.3389/fcvm.2019.00160 PMC6873287

[14] Henry A, Arnott C, Makris A, Davis G, Hennessy A, Beech A et al. Blood pressure Postpartum (Bp2) rct protocol: follow-up and Lifestyle Behaviour Change Strategies in the First 12 months after hypertensive pregnancy. Pregnancy Hypertens. 2020;22. 10.1016/j.preghy.2020.07.001 32679537

[15] Reimer MSG. Evaluation of practicability in Survey and Test procedures and of successful implementation of Complex interventions–feasibility analysis based on a pilot study: influence of conditional workout postpartum on arterial stiffness among women with Status after Preeclampsia, Superimposed Preeclampsia or Hellp-Syndrome [Registered Trial].

[16] Hauspurg A, Seely EW, Rich-Edwards J, Hayduchok C, Bryan S, Roche AT et al. Postpartum Home Blood Pressure Monitoring and Lifestyle Intervention in Overweight and Obese Individuals the First Year after Gestational Hypertension or Pre-Eclampsia: A Pilot Feasibility Trial. BJOG: Int J Obstet Gynaecol. 2023. 10.1111/1471-0528.17381 PMC10880812 36655365

[17] Hutchesson MJ, Taylor R, Shrewsbury VA, Vincze L, Campbell LE, Callister R, et al. Be Healthe for your heart: a pilot randomized controlled Trial evaluating a web-based behavioral intervention to improve the Cardiovascular Health of women with a history of Preeclampsia. Int J Environ Res Public Health. 2020;17(16):5779. 32785044 10.3390/ijerph17165779 PMC7459885

[18] Slater K, Taylor R, McLaughlin K, Pennell C, Collins C, Hutchesson M. Barriers and facilitators to Cardiovascular Disease Prevention following Hypertensive disorders of pregnancy in primary Care: cross-sectional surveys. Nutrients. 2023;15(17). 10.3390/nu15173817 PMC10490358 37686849

[19] Eysenbach G. Improving the quality of web surveys: the Checklist for reporting results of internet E-Surveys (Cherries). J Med Internet Res. 2004;6(3):e34. 15471760 10.2196/jmir.6.3.e34 PMC1550605

[20] von Elm E, Altman DG, Egger M, Pocock SJ, Gøtzsche PC, Vandenbroucke JP. The strengthening the reporting of Observational studies in Epidemiology (Strobe) Statement: guidelines for reporting observational studies. Lancet. 2007;370(9596):1453–7. 18064739 10.1016/S0140-6736(07)61602-X

[21] Australian Action on Preeclampsia. 2023 [ https://www.aapec.org.au/]

[22] Australasian Birth Trauma Association. 2023 [ https://birthtrauma.org.au/]

[23] MamaTribe. 2023 [ https://www.mamatribe.com.au/]

[24] Harris PA, Taylor R, Thielke R, Payne J, Gonzalez N, Conde JG. Research Electronic Data Capture (Redcap)—a Metadata-Driven methodology and workflow process for providing Translational Research Informatics support. J Biomed Inf. 2009;42(2):377–81. 10.1016/j.jbi.2008.08.010 PMC2700030 18929686

[25] Harris PA, Taylor R, Minor BL, Elliott V, Fernandez M, O’Neal L, et al. The Redcap Consortium: building an International Community of Software platform partners. J Biomed Inf. 2019;95:103208. 10.1016/j.jbi.2019.103208 PMC7254481 31078660

[26] Gresham E, Forder P, Chojenta CL, Byles JE, Loxton DJ, Hure AJ. Agreement between self-reported perinatal outcomes and Administrative Data in New South Wales, Australia. BMC Pregnancy Childbirth. 2015;15(1):161. 26238999 10.1186/s12884-015-0597-x PMC4524430

[27] Beekers P, Jamaladin H, van Drongelen J, Roeleveld N, van Gelder MMHJ. Data from web-based questionnaires were valid for gestational diabetes and Preeclampsia, but not gestational hypertension. J Clin Epidemiol. 2020;125:84–90. 32473198 10.1016/j.jclinepi.2020.05.023

[28] Australian Bureau of Statistics. Socio-Economic Indexes for Areas (Seifa), Canberra, Australia. 2021 [cited June 6 2023]. https://www.abs.gov.au/statistics/people/people-and-communities/socio-economic-indexes-areas-seifa-australia/latest-release

[29] StataCorp. Stata Statistical Software: release 16: Statacorp Llc. College Station, TX; 2019.

[30] Magee LA, Brown MA, Hall DR, Gupte S, Hennessy A, Karumanchi SA, et al. The 2021 International Society for the Study of Hypertension in Pregnancy Classification, Diagnosis & Management Recommendations for International Practice. Pregnancy Hypertens. 2022;27:148–69. 35066406 10.1016/j.preghy.2021.09.008

[31] Roth H, Morcos V, Roberts LM, Hanley L, Homer CSE, Henry A. Preferences of Australian Healthcare Providers Regarding Education on Long-Term Health after Hypertensive disorders of pregnancy: a qualitative study. BMJ Open. 2022;12(5):e055674. 35618327 10.1136/bmjopen-2021-055674 PMC9137339

[32] Roth H, Homer CSE, LeMarquand G, Roberts LM, Hanley L, Brown M, et al. Assessing Australian women’s knowledge and knowledge preferences about Long-Term Health after Hypertensive disorders of pregnancy: a Survey Study. BMJ Open. 2020;10(12):e042920–e. 33334841 10.1136/bmjopen-2020-042920 PMC7747529

[33] Atkinson J, Wei W, Potenza S, Simpson G, Middleton A, Walker S, et al. Patients’ understanding of Long-Term Cardiovascular risks and Associated Health-seeking behaviours after Pre-eclampsia. Open Heart. 2023;10(1):e002230. 36914205 10.1136/openhrt-2022-002230 PMC10016282

[34] Society of Obstetric Medicine Australia and New Zealand. Hypertension in Pregnancy Guideline, Sydney. 2023.

[35] Hutchesson M, Shrewsbury V, Park F, Callister R, Collins C. Are women with a recent diagnosis of Pre-eclampsia Aware of their Cardiovascular Disease Risk? A cross-sectional survey. Australian New Z J Obstet Gynecol. 2018;58(6):E27–8. 10.1111/ajo.12900 30536506

[36] Ju I, Banks E, Calabria B, Ju A, Agostino J, Korda RJ, et al. General practitioners’ perspectives on the Prevention of Cardiovascular Disease: systematic review and thematic synthesis of qualitative studies. BMJ Open. 2018;8(11):e021137. 30389756 10.1136/bmjopen-2017-021137 PMC6224770

[37] Filc D, Davidovich N, Novack L, Balicer RD. Is socioeconomic Status Associated with Utilization of Health Care Services in a single-payer Universal Health Care System? Int J Equity Health. 2014;13:115. 25431139 10.1186/s12939-014-0115-1 PMC4260253

[38] Turner LR, Cicuttini F, Pearce C, Mazza D. Cardiovascular Disease Screening in General Practice: General Practitioner Recording of Common Risk factors. Prev Med. 2017;99:282–5. 28322884 10.1016/j.ypmed.2017.03.004

[39] Heshmati A, Mishra G, Koupil I. Childhood and Adulthood Socio-Economic position and Hypertensive disorders in pregnancy: the Uppsala Birth Cohort Multigenerational Study. J Epidemiol Commun Health. 2013;67(11):939. 10.1136/jech-2012-202149 23729327

[40] Mattsson K, Juárez S, Malmqvist E. Influence of Socio-Economic factors and region of birth on the risk of Preeclampsia in Sweden. Int J Environ Res Public Health. 2022;19(7). 10.3390/ijerph19074080 PMC8998104 35409763

[41] Nicholls-Dempsey L, Badeghiesh A, Baghlaf H, Dahan MH. How does High Socioeconomic Status affect maternal and neonatal pregnancy outcomes? A Population-based study. Am J Obstet Gynecol. 2022;226(1):S452–3. 10.1016/j.eurox.2023.100248 PMC10590715 37876770

[42] Ray AE, Jeffrey KN, Nair PH, Vu QD, King F, Schmied V. You’re a ‘High-Risk’ customer: a qualitative study of women’s experiences of receiving information from Health professionals regarding health problems or complications in pregnancy. Women Birth. 2022;35(5):e477–86. 34974953 10.1016/j.wombi.2021.12.002

[43] Gamble D, Brikinns B, Myint P, Bhattacharya S. Hypertensive disorders of pregnancy and subsequent Cardiovascular Disease: current National and International guidelines and the need for Future Research. Front Cardiovasc Med. 2019;6(55). 10.3389/fcvm.2019.00055 PMC6533460 31157237

[44] Brown MA, Magee LA, Kenny LC, Karumanchi SA, McCarthy FP, Saito S, et al. The Hypertensive disorders of pregnancy: Isshp classification, Diagnosis & Management Recommendations for International Practice. Pregnancy Hypertens. 2018;13:291–310. 29803330 10.1016/j.preghy.2018.05.004

[45] Brodribb W, Zadoroznyj M, Dane A. The views of mothers and gps about Postpartum Care in Australian General Practice. BMC Fam Pract. 2013;14(1):139. 24066802 10.1186/1471-2296-14-139 PMC3851599

[46] Khosla K, Heimberger S, Nieman KM, Tung A, Shahul S, Staff AC, et al. Long-Term Cardiovascular Disease Risk in women after Hypertensive disorders of pregnancy: recent advances in hypertension. Hypertension. 2021;78(4):927–35. 34397272 10.1161/HYPERTENSIONAHA.121.16506 PMC8678921

