# Supplementary material for: Awareness of cardiovascular disease risk and care received among Australian women with a history of hypertensive disorders of pregnancy: a cross-sectional survey
Source: BMC Pregnancy Childbirth. 2025 Jan 8;25:15. doi: 10.1186/s12884-024-07018-5 (PMC11708001; doi:10.1186/s12884-024-07018-5)
Supplement: Supplementary file 1 — Supplementary Material 1 [file 12884_2024_7018_MOESM1_ESM.docx]

## Additional Files 1

**Survey: Understanding current practice and guidelines, along with barriers and facilitators to heart disease primary and secondary prevention following high blood pressure in pregnancy.**

**
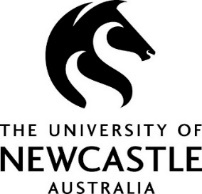
**

**About the survey**

A team of researchers from the University of Newcastle want your help to understand the health care journey of women following a diagnosis of a high blood pressure problem during pregnancy, such as chronic hypertension, gestational hypertension, or preeclampsia.

They are interested in finding out from Australian women who have had chronic hypertension, gestational hypertension and/or preeclampsia:

- Whether they have received any health care for prevention or risk factor screening of heart disease.
- What influences whether they have or have not received health care for prevention or risk factor screening of heart disease.
- What might help them receive better health care for prevention or regular risk factor screening of heart disease.

***Who can take part in the survey?***

We are seeking women over 18 years of age, who live in Australia and were diagnosed by a health professional from 2017 onwards as having a high blood pressure problem during a pregnancy, to participate in a short 10-minute survey. Those who complete the survey will also go into a prize draw to win **one of 10 $200** **gift cards**, to be used at select clothing, beauty, homeware stores around Australia and online!

### *Want to know more about the survey?*

### Click on this Information Statement

### *Please read this Information Statement before proceeding to the survey below.*

***Complaints about this research***

This project has been approved by the University’s Human Research Ethics Committee, Approval No. H-2021-0415. Should you have concerns about your rights as a participant in this research, or you have a complaint about the manner in which the research is conducted, it may be given to the researcher, or, if an independent person is preferred, to the Ethics Officer, Research and Innovation Services, The University of Newcastle, University Drive, Callaghan NSW 2308, Australia, telephone (02) 4921 6333 or email [Human-Ethics@newcastle.edu.au](mailto:Human-Ethics@newcastle.edu.au).

|  | Do you wish to participate in this survey? | | |
| --- | --- | --- | --- |
|  | Note: Selecting yes below will be taken as your informed consent to participate. | | |
|  | | Yes |  |
|  | | No |  |

**Eligibility screening (new page)**

1. What is your age in years?

| Only ages over 18 will be available. |
| --- |

1. In how many pregnancies, since 2017, have you been diagnosed by a health professional as having the following:

**Chronic hypertension (also called essential hypertension):** is high blood pressure (also known as hypertension) before falling pregnant, outside of pregnancy, or in the first half of your pregnancy (before 20 weeks gestation).

**Gestational hypertension:** is high blood pressure diagnosed in the second half of your pregnancy (after 20 weeks of pregnancy) with **no** additional health issues such as protein in your urine, liver, kidney problems, or concerns about the growth of your baby while you were pregnant.

**Preeclampsia:** is high blood pressure in the second half of your pregnancy (after 20 weeks of pregnancy) and some additional health signs or issues in you and/or your baby (e.g., protein in your urine, liver or kidney problems, or concerns about the growth of your baby while you were pregnant).

| **Statements** | **None** | **1** | **2** | **3** | **4** | **5** |
| --- | --- | --- | --- | --- | --- | --- |
| 1. Chronic hypertension | 0 | 1 | 2 | 3 | 4 | 5 |
| 1. Gestational hypertension | 0 | 1 | 2 | 3 | 4 | 5 |
| 1. Preeclampsia | 0 | 1 | 2 | 3 | 4 | 5 |

1. In what year were you most recently diagnosed with chronic hypertension, gestational hypertension and/or preeclampsia?

| 1. 2017 | 1 |
| --- | --- |
| 1. 2018 | 2 |
| 1. 2019 | 3 |
| 1. 2020 | 4 |
| 1. 2021 | 5 |
| 1. None of the above (survey to end) | 6 |

1. Do you currently live in Australia?

| 1. Yes | 1 |
| --- | --- |
| 1. No (survey to end) | 2 |

**The following questions are about your pregnancies and healthcare after pregnancy (new page)**

1. What type of blood pressure problem were you diagnosed with in your most recent pregnancy effected by hypertension? (You may select more than one).

| 1. Chronic hypertension | 1 |
| --- | --- |
| 1. Gestational hypertension | 2 |
| 1. Preeclampsia | 3 |

1. How many weeks in your most recent pregnancy that you experienced a high blood pressure problem did your health professional diagnosed you with ___________ (will automatically input whatever they have chosen before)?

| 1. 20-28 weeks | 1 |
| --- | --- |
| 1. 28-34 weeks | 2 |
| 1. 34-37 weeks | 3 |
| 1. 37-40 weeks | 4 |
| 1. 40-42 weeks | 5 |
| 1. During or after giving birth | 6 |
| 1. I cannot remember | 7 |
| 1. Other, please specify ___________________________ | 8 |

1. What is the date of birth for your child born in your most recent pregnancy complicated by a hypertensive disorder of pregnancy?

| DD/MM/YYYY |
| --- |

1. In your most recent pregnancy with _________, which health professional/s cared for you? (You can select more than one)

| 1. General practitioner | 1 |
| --- | --- |
| 1. Private obstetrician | 2 |
| 1. Public obstetrician | 3 |
| 1. Private midwife | 4 |
| 1. Public midwife | 5 |
| 1. Other, please specify ___________________________ | 6 |

**The following questions are asking you about the care you have received from health professionals since the time of your most recent pregnancy that was complicated by a high blood pressure problem (identified above).**

1. Before reading the information about this survey, were you aware that after experiencing a high blood pressure problem in pregnancy you have a greater risk of developing heart disease?

| 1. Yes (go to 10) | 1 |
| --- | --- |
| 1. No (go to 11) | 2 |

1. How were you made aware of this? (You may select more than one)

| 1. General practitioner (go to 11) | 1 |
| --- | --- |
| 1. Obstetrician (go to 11) | 2 |
| 1. Midwife (go to 11) | 3 |
| 1. My own research (go to 12) | 4 |
| 1. Talking to family/friends that experienced preeclampsia or gestational hypertension (go to 12) | 5 |
| 1. Support groups (i.e., Facebook group) (go to 12) | 6 |
| 1. Other, please specify ___________________________ (go to 12) | 7 |

1. When did your health professional discuss for future risk of heart disease with you?

| 1. Before birth | 1 |
| --- | --- |
| 1. Immediately after birth | 2 |
| 1. Within the first 6 weeks | 3 |
| 1. 6 weeks to 6 months | 4 |
| 1. 6 months to one year | 5 |
| 1. 1 year or more | 6 |
| 1. I cannot remember | 7 |
| 1. A health professional has not discussed the risk of future health conditions with me. | 8 |
| 1. Other __________________________________________ | 9 |

1. When do you think would be a good time to receive information about long term health risks after a pregnancy effected by a blood pressure problem (in months)?

| Sliding scale from during pregnancy/after birth to 24 months after pregnancy. |
| --- |

1. Did you attend a 6-week postpartum appointment with a health professional?

| 1. Yes (go to 14) | 1 |
| --- | --- |
| 1. No (go to 15) | 2 |

1. What type of health professional did you see at this 6-week postpartum appointment?

| 1. General practitioner | 1 |
| --- | --- |
| 1. Obstetrician | 2 |
| 1. Midwife | 3 |
| 1. Other, please specify ___________________________ | 4 |

1. Has a health professional/s asked about or provided advice for management of the following: Please select all that apply.

| 1. Your health during past pregnancies | 1 |
| --- | --- |
| 1. Blood pressure | 2 |
| 1. Blood lipids (cholesterol studies) | 3 |
| 1. Blood glucose levels and/or insulin | 4 |
| 1. None of the above | 5 |

1. Which health professional/s? Please select all that apply. (If answered yes to any of Q15 a-d, if not they will go straight to Q17).

| 1. General Practitioner | 1 |
| --- | --- |
| 1. Obstetrician | 2 |
| 1. Midwife | 3 |
| 1. Other, please specify _______________________ | 4 |

1. Has a health professional/s asked about or provided advice for management of the following after a pregnancy effected by high blood pressure: Please select all that apply.

| 1. Diet/healthy eating | 1 |
| --- | --- |
| 1. Physical activity | 2 |
| 1. Smoking cessation | 3 |
| 1. Alcohol consumption | 4 |
| 1. Sleep habits | 5 |
| 1. Weight management | 6 |
| 1. Mental health and/or stress management | 7 |
| 1. None of the above | 8 |

1. Which health professional/s? Please select all that apply. (If answered yes to any of Q17 a-g, if not they will go straight to Q19).

| 1. General Practitioner | 1 |
| --- | --- |
| 1. Obstetrician | 2 |
| 1. Midwife | 3 |
| 1. Allied health professional (e.g., dietitian, psychologist, physiotherapist) | 4 |
| 1. Other, please specify _______________________ | 5 |

**The following questions are asking you about the care you have received from a general practitioner since the time of your most recent pregnancy that was complicated by a high blood pressure problem (identified above), as well as general barriers that you may face when accessing care from a general practitioner.**

1. Were you encouraged to make an appointment with your general practitioner after pregnancy?

| 1. Yes | 1 |
| --- | --- |
| 1. No | 2 |

1. How many times per year since your most recent pregnancy complicated by a high blood pressure problem have you visited a general practitioner for yourself?

| 1. ≥ 20 times | 1 |
| --- | --- |
| 1. 12-19 times | 2 |
| 1. 6-11 times | 3 |
| 1. 4-5 times | 4 |
| 1. 1-3 times | 5 |
| 1. None | 6 |

1. If you are to visit a general practitioner, what would be your main reason for the visit?

| 1. Acute illness (appears suddenly and lasts for a short time) e.g., common cold, flu. | 1 |
| --- | --- |
| 1. Respiratory illness e.g., asthma | 2 |
| 1. Circulatory illness e.g., heart disease | 3 |
| 1. Endocrine conditions e.g., diabetes, polycystic ovarian syndrome | 4 |
| 1. Mental health (e.g., psychological) | 5 |
| 1. Preventative care e.g., weight management | 6 |
| 1. Skin check/care | 7 |
| 1. Pregnancy and family planning | 8 |
| 1. Digestive concerns | 9 |
| 1. Musculoskeletal e.g., arthritis, back pain, gout | 10 |
| 1. Other ________________________________________ | 11 |

1. What factors, if any, discourage you from seeing the general practitioner? (You may select more than one).

| 1. Cost | 1 |
| --- | --- |
| 1. Time commitment | 2 |
| 1. Lack of childcare facilities | 3 |
| 1. Lack of understanding of available services | 4 |
| 1. Opening hours of the service | 5 |
| 1. Transport | 6 |
| 1. Language barriers | 7 |
| 1. Cultural or religious barriers | 8 |
| 1. Difficulty getting an appointment | 9 |
| 1. Other ____________________________________________ | 10 |

1. When you see a general practitioner, do you feel satisfied by their **interest** in **how you feel** about the treatment or advice they provide?

| 1. Yes | 1 |
| --- | --- |
| 1. No | 2 |

1. When you see a general practitioner, do you feel satisfied by their **explanation** of your problem and/or the treatment they provided?

| 1. Yes | 1 |
| --- | --- |
| 1. No | 2 |

1. When seeing a general practitioner for your own care, how often would you go to discuss preventative health care (e.g., lifestyle behaviours such as diet, smoking cessation)?

| 1. Always | 1 |
| --- | --- |
| 1. Often | 2 |
| 1. Sometimes | 3 |
| 1. Occasionally | 4 |
| 1. Never | 5 |

1. When you see a general practitioner, do you feel you have **enough time** to discuss strategies to improve your heart health?

| 1. Yes | 1 |
| --- | --- |
| 1. No | 2 |

1. Has a general practitioner directed you to resources for heart disease prevention e.g., the Heart Foundation website?

| 1. Yes (go to 28) | 1 |
| --- | --- |
| 1. No (go to 30) | 2 |

1. Can you please elaborate on what resources the general practitioner has provided you with?

| Free text (go to 29) |
| --- |

1. Did you attend a follow-up appointment with your general practitioner after receiving heart disease advice and/or resources?

| 1. Yes | 1 |
| --- | --- |
| 1. No | 2 |

1. Do you feel supported by a general practitioner if you were to implement any of the lifestyle changes provided? Please select from the following, you can select more than one.

| 1. Dietary intake/healthy eating | Yes | No |
| --- | --- | --- |
| 1. Physical activity | Yes | No |
| 1. Smoking | Yes | No |
| 1. Alcohol intake | Yes | No |
| 1. Sleep habits | Yes | No |
| 1. Weight management | Yes | No |
| 1. Mental health and/or stress management | Yes | No |

1. Can you please elaborate on why/how you feel supported to implement the lifestyle change/s you chose above? (If they select no to all, this question will not come up and they will go straight to 32).

| Free text (go to 32) |
| --- |

**The following questions will help researchers understand what makes it harder and what makes it easier for you to obtain care from general practitioners when it comes to heart disease prevention after a blood pressure problem during pregnancy (new page).**

1. Out of the following, what could GPs do to provide the best support to you to help improve your heart health, which do you believe are the top priorities? *You may pick up to 3 priorities.*

| **Potential strategies** |  |
| --- | --- |
| 1. Provide the appropriate assessment and advice regarding heart disease. | 1 |
| 1. Provide you with resources (printed or digital) to take home after consultations, explaining how to reduce your risk of heart disease. | 2 |
| 1. Refer you to a specific health professional for advice to reduce your risk of heart disease e.g., a dietitian for advice with dietary intake. | 3 |
| 1. Improve their communication with your other health professionals, so that your care team are all aware of your obstetric history and future long-term health. | 4 |
| 1. Communicate to you the long-term effects of a hypertensive disorder of pregnancy and what that means for your health care. | 5 |
| 1. Provide you with ongoing follow-up of the assessment and advice they provide regarding heart disease. | 6 |
| 1. Provide you with pre-pregnancy planning advice, discussing potential pregnancy complications and what to inform them on during follow up. | 7 |
| 1. Using a checklist of questions to ask women during their first postpartum appointment. | 8 |
| 1. I do not think any of these strategies would be useful (none of the above) (go to 34) | 9 |

1. Please elaborate on why you have chosen the above strategy/s? (go to 35)

| Free text |
| --- |

1. Are there any other strategies that general practitioners’ could implement to provide the best support to you to help improve your heart health?

| Free text |
| --- |

**These last seven questions are to find out about you. These demographic questions will ensure that when informing interventions for heart disease prevention after blood pressure problems during pregnancy, we are targeting the correct population and tailoring it appropriately.**

1. What is your present marital/relationship status?

| 1. Single | 1 |
| --- | --- |
| 1. De facto | 2 |
| 1. Married | 3 |
| 1. Divorced | 4 |
| 1. Separated, but not divorced | 5 |
| 1. Married | 6 |

1. Are you of Aboriginal or Torres Strait Islander origin?

*For persons of both Aboriginal and Torres Strait Islander origin, mark both ‘Yes’ boxes.*

| 1. No | 1 |
| --- | --- |
| 1. Yes, Aboriginal | 2 |
| 1. Yes, Torres Strait Islander | 3 |

1. In which country were you born?

| 1. Australia | 1 |
| --- | --- |
| 1. New Zealand | 2 |
| 1. England | 3 |
| 1. United states of America | 4 |
| 1. Canada | 5 |
| 1. India | 6 |
| 1. Italy | 7 |
| 1. Vietnam | 8 |
| 1. Philippines | 9 |
| 1. Other ___________________________________________ | 10 |

1. Do you usually speak a language other than English at home? *If more than one language other than English, select the one that is spoken most often.*

| 1. No, I only speak English at home | 1 |
| --- | --- |
| 1. Yes, Mandarin | 2 |
| 1. Yes, Italian | 3 |
| 1. Yes, Arabic | 4 |
| 1. Yes, Cantonese | 5 |
| 1. Yes, Greek | 6 |
| 1. Yes, Vietnamese | 7 |
| 1. Yes, Other _______________________________________ | 8 |

1. What is the postcode where you currently live? ___________________________________
2. What is your current household income?

*Household income is the combined total gross income of all members of a household above 15 years of age.* *Income includes wages and salaries; government pensions, benefits and allowances; profit or losses from business or investments; other sources of income.* *Do not deduct: Tax, superannuation contributions, amounts salary sacrificed, or any other automatic deductions.*

| 1. $3,000 or more per week $156,000 or more per year | 1 |
| --- | --- |
| 1. $2,000 - $2,999 per week $104,000 - $155,999 per year | 2 |
| 1. $1,750 - $1,999 per week $91,000 - $103,999 per year | 3 |
| 1. $1,500 - $1,749 per week $78,000 - $90,999 per year | 4 |
| 1. $1,250 - $1,499 per week $65,000 - $77,999 per year | 5 |
| 1. $1,000 - $1,249 per week $52,000 - $64,999 per year | 6 |
| 1. $800 - $999 per week $41,600 - $51,999 per year | 7 |
| 1. $650 - $799 per week $33,800 - $41,599 per year | 8 |
| 1. $500 - $649 per week $26,000 - $33,799 per year | 9 |
| 1. $400 - $499 per week $20,800 - $25,999 per year | 10 |
| 1. $300 - $399 per week $15,600 - $20,799 per year | 11 |
| 1. $150 - $299 per week $7,800 - $15,599 per year | 12 |
| 1. $1 - $149 per week $1 - $7,799 per year | 13 |
| 1. Nil income | 14 |
| 1. Negative income` | 15 |
| 1. Don’t know | 16 |
| 1. Don’t want to answer | 17 |
| 1. I live alone (house income is the same as my income) | 18 |

1. What is your highest level of education?

| 1. No formal qualifications | 1 |
| --- | --- |
| 1. School Certificate (Year 10 or equivalent) | 2 |
| 1. High School Certificate (Year 12 or equivalent) | 3 |
| 1. Trade/Apprenticeship (e.g., Hairdresser, Chef) | 4 |
| 1. Certificate/Diploma (e.g., Childcare, Technician) | 5 |
| 1. University Degree (e.g., Bachelor’s Degree) | 6 |
| 1. Higher University Degree (e.g., Grad Dip, Masters, PhD) | 7 |

**Additional Table 1. Demographic characteristics of participants by HDP type**

| **Subtype of HDP (total n=293)** | **Chronic Hypertension**  **(n=16)** | **Gestational Hypertension**  **(n=104)** | **Preeclampsia (n=173)** | **P-value** |
| --- | --- | --- | --- | --- |
| **Variables** | **Value** | | |  |
| Age | Mean (SD) | | |  |
|  | 33.40 (4.81) | 32.79 (4.72) | 33.76 (4.87) | 0.94 |
| **Demographic characteristics** | **Total n (%)** | | |  |
| **Marital status** | | | | 0.49 |
| Single | 0 (0) | 0 (0) | 6 (4) |  |
| De facto | 2 (13) | 18 (17) | 39 (23) |  |
| Married | 14 (88) | 85 (82) | 126 (73) |  |
| Divorced/separated | 0 (0) | 1 (1) | 2 (1) |  |
| **Country/area of birth** | | | | 0.04 |
| Australia/New Zealand | 15 (94) | 98 (94) | 158 (91) |  |
| North America (USA and Canada) | 1 (6) | 0 (0) | 1 (1) |  |
| Central/South America | 0 (0) | 1 (1) | 2 (1) |  |
| United Kingdom/Ireland | 0 (0) | 3 (3) | 5 (3) |  |
| Asia | 0 (0) | 1 (1) | 6 (4) |  |
| Africa/South Africa | 2 (1) | 1 (1) | 1 (1) |  |
| **State of residence** | | | | 0.25 |
| New South Wales | 8 (50) | 50 (48) | 88 (51) |  |
| Victoria | 5 (31) | 29 (28) | 35 (20) |  |
| Queensland | 1 (6) | 10 (10) | 26 (15) |  |
| South Australia | 0 (0) | 0 (0) | 9 (5) |  |
| Australian Capital Territory | 1 (6) | 4 (4) | 5 (3) |  |
| Northern Territory | 0 (0) | 1 (1) | 2 (1) |  |
| Western Australia | 1 (6) | 7 (7) | 8 (5) |  |
| Tasmania | 0 (0) | 3 (3) | 0 (0) |  |
| **Socio Economic Indexes for Areas (SEIFA) Quintiles** | | | | 0.27 |
| Quintile 1 | 0 (0) | 8 (8) | 19 (11) |  |
| Quintile 2 | 5 (31) | 18 (17) | 28 (16) |  |
| Quintile 3 | 4 (25) | 29 (28) | 58 (34) |  |
| Quintile 4 | 3 (19) | 30 (29) | 36 (21) |  |
| Quintile 5 | 4 (25) | 19 (18) | 32 (19) |  |
| **Highest level of education** | | | | 0.33 |
| No formal qualifications | 0 (0) | 1 (1) | 0 (0) |  |
| School Certificate (Year 10 or equivalent) | 1 (6) | 4 (4) | 4 (2) |  |
| High school Certificate (Year 12 or equivalent) | 3 (19) | 2 (2) | 15 (9) |  |
| Trade/Apprenticeship | 0 (0) | 3 (3) | 4 (2) |  |
| Certificate/Diploma | 2 (13) | 21 (20) | 39 (23) |  |
| University undergraduate degree | 5 (31) | 49 (47) | 70 (41) |  |
| Higher university degree (postgraduate) | 5 (31) | 24 (23) | 41 (24) |  |
| **Household income** | | | | 0.31 |
| $3,000 + per week or $156,000 per year or more | 3 (19) | 17 (16) | 39 (23) |  |
| $2,000 - $2,999 per week or $104,000 $155,999 per year | 3 (19) | 16 (15) | 31 (18) |  |
| $1,750 - $1,999 per week $91,000 - $103,999 per year | 3 (19) | 35 (34) | 38 (22) |  |
| $1,500 - $1,749 per week $78,000 - $90,999 per year | 1 (6) | 12 (12) | 25 (14) |  |
| $1,250 - $1,499 per week $65,000 - $77,999 per year | 2 (13) | 5 (5) | 14 (8) |  |
| $1,000 - $1,249 per week $52,000 - $64,999 per year | 1 (6) | 8 (8) | 11 (6) |  |
| $800 - $999 per week $41,600 - $51,999 per year | 3 (19) | 1 (1) | 9 (5) |  |
| $650 - $799 per week $33,800 - $41,599 per year or less | 0 (0) | 5 (5) | 4 (2) |  |
| Don’t want to answer | 0 (0) | 5 (5) | 2 (1) |  |
| **Most recent diagnosis of HDP*** | | | | 0.48 |
| 2017 | 1 (6) | 7 (7) | 14 (8) |  |
| 2018 | 4 (25) | 7 (7) | 19 (11) |  |
| 2019 | 1 (6) | 16 (15) | 23 (13) |  |
| 2020 | 5 (31) | 28 (27) | 38 (22) |  |
| 2021 onwards | 5 (31) | 46 (44) | 79 (46) |  |
| **Type of health professional involved in care during pregnancy** | | | |  |
| General practitioner | 9 (56) | 25 (24) | 55 (32) | 0.03 |
| **Obstetrician**  Public  Private | 5 (31)  8 (50) | 36 (35)  47 (45) | 76 (44)  68 (39) | 0.24  0.50 |
| **Midwife**  Public  Private | 5 (31)  2 (13) | 48 (46)  7 (7) | 81 (47)  12 (7) | 0.49  0.70 |

*HDP: hypertensive disorder of pregnancy. SEIFA: Socioeconomic Indexes for Areas are quintiles/codes that indicate socioeconomic status. 1=lowest socioeconomic areas and 5=highest socioeconomic areas.

**Additional Table 2. Demographic characteristics of participants by SES**

| **Subtype of HDP** | **Quintile 1 (n=26)** | **Quintile 2**  **(n=52)** | **Quintile 3 (n=89)** | **Quintile 4 (n=72)** | **Quintile 5 (n=54)** | **P-value** |
| --- | --- | --- | --- | --- | --- | --- |
| **Variables** | **Value** | | | | | |
| Age | Mean (SD) | | | | | |
|  | 31.79 (4.94) | 33.16 (4.91) | 33.92 (4.93) | 32.57 (4.55) | 34.90 (4.47) | 0.90 |
| **Demographic characteristics** | **Total n (%)** | | | | | |
| **Marital status** | | | | | | 0.80 |
| Single | 0 (0) | 1 (2) | 2 (2) | 1 (2) | 2 (3) |  |
| De facto | 7 (24) | 13 (23) | 15 (17) | 12 (18) | 12 (20) |  |
| Married | 21 (72) | 41 (73) | 72 (80) | 55 (81) | 45 (76) |  |
| Divorced/separated | 0 (0) | 0 (0) | 1 (1) | 0 (0) | 0 (0) |  |
| **Country/area of birth** | | | | | | 0.12 |
| Australia/New Zealand | 26 (90) | 54 (96) | 85 (94) | 60 (88) | 55 (93) |  |
| North America (USA and Canada) | 0 (0) | 2 (4) | 0 (0) | 0 (0) | 0 (0) |  |
| Central/South America | 1 (3) | 0 (0) | 1 (1) | 1 (2) | 0 (0) |  |
| United Kingdom/Ireland | 1 (4) | 0 (0) | 2 (2) | 3 (4) | 2 (3) |  |
| Asia | 0 (0) | 0 (0) | 1 (1) | 3 (4) | 2 (3) |  |
| Africa/South Africa | 1 (4) | 0 (0) | 1 (1) | 0 (0) | 0 (0) |  |
| **State of residence** | | | | | | <0.00 |
| New South Wales | 14 (48) | 40 (71) | 58 (64) | 25 (37) | 13 (22) |  |
| Victoria | 6 (21) | 8 (14) | 18 (20) | 20 (29) | 20 (34) |  |
| Queensland | 6 (21) | 5 (9) | 10 (11) | 10 (15) | 8 (14) |  |
| South Australia | 2 (7) | 0 (0) | 3 (3) | 1 (2) | 3 (5) |  |
| Australian Capital Territory | 0 (0) | 0 (0) | 0 (0) | 1 (2) | 9 (15) |  |
| Northern Territory | 1 (4) | 0 (0) | 0 (0) | 0 (0) | 2 (3) |  |
| Western Australia | 0 (0) | 1 (2) | 1 (1) | 11 (16) | 3 (5) |  |
| Tasmania | 0 (0) | 2 (4) | 0 (0) | 0 (0) | 1 (2) |  |
| **Type of Hypertensive Disorder of Pregnancy** | | | | | | 0.35 |
| Chronic Hypertension | 0 (0) | 5 (9) | 4 (5) | 3 (5) | 4 (7) |  |
| Gestational Hypertension | 8 (28) | 18 (33) | 29 (33) | 30 (46) | 19 (33) |  |
| Preeclampsia | 15 (52) | 26 (48) | 49 (56) | 28 (42) | 29 (51) |  |
| Gestational Hypertension and Preeclampsia | 6 (21) | 5 (9) | 5 (6) | 5 (8) | 5 (9) |  |
| **Highest level of education** | | | | | | 0.59 |
| No formal qualifications | 0 (0) | 0 (0) | 1 (1) | 0 (0) | 0 (0) |  |
| School Certificate (Year 10 or equivalent) | 0 (0) | 3 (5) | 3 (3) | 2 (3) | 1 (2) |  |
| High school Certificate (Year 12 or equivalent) | 3 (10) | 7 (13) | 4 (4) | 4 (6) | 3 (5) |  |
| Trade/Apprenticeship | 1 (4) | 2 (4) | 3 (3) | 1 (2) | 0 (0) |  |
| Certificate/Diploma | 10 (35) | 12 (21) | 20 (22) | 17 (25) | 8 (14) |  |
| University undergraduate degree | 9 (31) | 19 (34) | 39 (43) | 31 (46) | 26 (44) |  |
| Higher university degree (postgraduate) | 6 (21) | 13 (23) | 20 (22) | 13 (19) | 21 (36) |  |
| **Household income** | | | | | | 0.05 |
| $3,000 + per week or $156,000 per year or more | 2 (7) | 9 (16) | 16 (18) | 14 (21) | 19 (32) |  |
| $2,000 - $2,999 per week or $104,000 $155,999 per year | 4 (14) | 6 (11) | 17 (19) | 16 (24) | 10 (17) |  |
| $1,750 - $1,999 per week $91,000 - $103,999 per year | 5 (17) | 21 (38) | 19 (21) | 20 (29) | 13 (22) |  |
| $1,500 - $1,749 per week $78,000 - $90,999 per year | 4 (14) | 6 (11) | 15 (17) | 11 (16) | 4 (7) |  |
| $1,250 - $1,499 per week $65,000 - $77,999 per year | 5 (17) | 6 (11) | 9 (10) | 0 (0) | 1 (2) |  |
| $1,000 - $1,249 per week $52,000 - $64,999 per year | 2 (7) | 3 (5) | 7 (8) | 5 (7) | 0 (0) |  |
| $800 - $999 per week $41,600 - $51,999 per year | 2 (7) | 4 (7) | 3 (3) | 1 (2) | 0 (0) |  |
| $650 - $799 per week $33,800 - $41,599 per year or less | 3 (10) | 1 (2) | 2 (2) | 1 (2) | 2 (3) |  |
| Don’t want to answer | 2 (7) | 0 (0) | 2 (2) | 0 (0) | 3 (5) |  |
| **Most recent diagnosis of HDP*** | | | | | | 0.06 |
| 2017 | 1 (3) | 3 (5) | 8 (9) | 4 (6) | 8 (14) |  |
| 2018 | 1 (3) | 9 (16) | 10 (11) | 4 (6) | 7 (12) |  |
| 2019 | 3 (10) | 10 (18) | 12 (13) | 4 (6) | 12 (20) |  |
| 2020 | 7 (24) | 9 (16) | 24 (27) | 25 (37) | 8 (14) |  |
| 2021 onwards | 17 (59) | 25 (45) | 36 (40) | 31 (46) | 24 (41) |  |
| **Type of health professional involved in care during pregnancy** | | | | | |  |
| General practitioner | 11 (38) | 9 (16) | 28 (31) | 20 (29) | 23 (39) | 0.08 |
| **Obstetrician**  Public  Private | 15 (52)  7 (24) | 17 (30)  24 (43) | 38 (42)  36 (40) | 25 (37)  33 (49) | 28 (48)  26 (44) | 0.23  0.26 |
| **Midwife**  Public  Private | 16 (55)  1 (4) | 27 (48)  5 (9) | 47 (52)  5 (6) | 28 (41)  5 (8) | 19 (32)  6 (10) | 0.11  0.74 |

*HDP: hypertensive disorder of pregnancy. SEIFA: Socioeconomic Indexes for Areas are quintiles/codes that indicate socioeconomic status. 1=lowest socioeconomic areas and 5=highest socioeconomic areas.

**Additional Table 3. Awareness of CVD risks, CVD risk marker and lifestyle CVD risk factor assessment by a health professional, by HDP type**

|  | **Chronic hypertension (n=16)** | **Gestational Hypertension**  **(n=104)** | **Preeclampsia (n=173)** | **p-value** |
| --- | --- | --- | --- | --- |
| **Awareness of CVD risk (n=293)** | 5 (31) | 22 (21) | 67 (39) | 0.01 |
| **CVD risk markers assessed by a health professional (n=293)** | **Total n (%)** | | |  |
| Obstetric history | 14 (88) | 84 (81) | 114 (66) | 0.01 |
| Blood pressure | 16 (100) | 83 (80) | 137 (79) | 0.13 |
| Blood lipids | 6 (38) | 31 (30) | 47 (27) | 0.65 |
| Blood glucose/insulin | 9 (56) | 43 (41) | 61 (35) | 0.20 |
| **Lifestyle CVD risk factors assessed by a health professional (n=293)** | **Total n (%)** | | |  |
| Diet | 7 (44) | 59 (57) | 82 (47) | 0.28 |
| Physical activity | 9 (56) | 71 (68) | 92 (53) | 0.05 |
| Smoking status | 8 (50) | 52 (50) | 76 (44) | 0.59 |
| Alcohol consumption | 8 (50) | 50 (48) | 76 (44) | 0.75 |
| Sleep habits | 7 (44) | 47 (45) | 78 (45) | 0.99 |
| Body weight/ overweight and obesity | 6 (38) | 44 (42) | 61 (35) | 0.50 |
| Stress/mental health | 13 (81) | 80 (77) | 130 (75) | 0.84 |

CVD: cardiovascular disease. HDP: hypertensive disorders of pregnancy

**Additional Table 4. Awareness of CVD risks, CVD risk marker and lifestyle CVD risk factor assessment by a health professional, by SES**

| **SEIFA Quintile** | **Quintile 1 (n=26)** | **Quintile 2**  **(n=52)** | **Quintile 3 (n=89)** | **Quintile 4 (n=72)** | **Quintile 5 (n=54)** | **P-value** |
| --- | --- | --- | --- | --- | --- | --- |
| **Awareness of CVD risk (n=293)** | **Total n (%)** | | | | | |
|  | 11 (42) | 16 (31) | 26 (29) | 22 (31) | 19 (35) | 0.75 |
| **CVD risk markers assessed by a health professional (n=293)** | **Total n (%)** | | | | | |
| Obstetric history | 15 (58) | 41 (79) | 68 (76) | 51 (71) | 37 (69) | 0.23 |
| Blood pressure | 20 (77) | 45 (87) | 71 (80) | 57 (79) | 43 (80) | 0.82 |
| Blood lipids | 6 (23) | 16 (31) | 24 (27) | 20 (28) | 18 (32) | 0.87 |
| Blood glucose/insulin | 9 (35) | 25 (48) | 33 (37) | 27 (38) | 19 (35) | 0.64 |
| **Lifestyle CVD risk factors assessed by a health professional (n=293)** | **Total n (%)** | | | | | |
| Diet | 13 (50) | 27 (52) | 47 (53) | 41 (57) | 20 (37) | 0.25 |
| Physical activity | 14 (54) | 33 (64) | 51 (57) | 46 (64) | 28 (52) | 0.62 |
| Smoking status | 11 (42) | 26 (50) | 46 (52) | 32 (44) | 21 (39) | 0.60 |
| Alcohol consumption | 11 (42) | 27 (52) | 44 (49) | 33 (46) | 19 (35) | 0.43 |
| Sleep habits | 12 (46) | 22 (42) | 39 (44) | 37 (51) | 22 (41) | 0.77 |
| Body weight/ overweight and obesity | 11 (42) | 20 (39) | 34 (38) | 31 (43) | 15 (28) | 0.50 |
| Stress/ mental health | 20 (77) | 43 (83) | 69 (78) | 55 (76) | 36 (67) | 0.41 |

CVD: cardiovascular disease. SES: socioeconomic status. SEIFA: Socioeconomic Indexes for Areas are quintiles/codes that indicate socioeconomic status. 1=lowest socioeconomic areas and 5=highest socioeconomic areas.

**Additional Table 5. Level of support women with a history of HDP perceived from general practitioners to make changes to lifestyle cardiovascular disease risk factors, by HDP type**

| **Subtype of HDP** | **Chronic Hypertension**  **(n=34)** | **Gestational Hypertension**  **(n=140)** | **Preeclampsia (n=173)** | **P-value** |
| --- | --- | --- | --- | --- |
| **CVD lifestyle risk markers*** | **Total n (%)** | | | |
| **Diet** | | | | 0.41 |
| Very supported | 4 (25) | 29 (28) | 39 (23) |  |
| Somewhat supported | 6 (38) | 26 (25) | 34 (20) |  |
| Neutral | 3 (19) | 26 (25) | 48 (28) |  |
| Not very supported | 1 (6) | 10 (10) | 23 (13) |  |
| Not at all supported | 1 (6) | 7 (7) | 6 (4) |  |
| **Physical activity** | | | | 0.50 |
| Very supported | 3 (19) | 24 (23) | 37 (21) |  |
| Somewhat supported | 5 (31) | 30 (29) | 34 (20) |  |
| Neutral | 5 (31) | 25 (24) | 47 (27) |  |
| Not very supported | 2 (13) | 13 (13) | 25 (14) |  |
| Not at all supported | 0 (0) | 6 (6) | 6 (4) |  |
| **Smoking status** | | | | 0.18 |
| Very supported | 1 (6) | 15 (14) | 19 (11) |  |
| Somewhat supported | 2 (13) | 9 (9) | 10 (6) |  |
| Neutral | 3 (19) | 8 (8) | 20 (12) |  |
| Not very supported | 2 (13) | 1 (1) | 5 (3) |  |
| Not at all supported | 0 (0) | 5 (5) | 4 (2) |  |
| **Alcohol consumption** | | | | 0.65 |
| Very supported | 2 (13) | 17 (16) | 24 (14) |  |
| Somewhat supported | 2 (13) | 14 (14) | 13 (8) |  |
| Neutral | 3 (19) | 12 (12) | 28 (16) |  |
| Not very supported | 2 (13) | 3 (3) | 9 (5) |  |
| Not at all supported | 0 (0) | 4 (4) | 5 (3) |  |
| **Sleep habits** | | | | 0.30 |
| Very supported | 1 (6) | 17 (16) | 35 (20) |  |
| Somewhat supported | 2 (13) | 25 (24) | 30 (17) |  |
| Neutral | 6 (38) | 23 (22) | 42 (24) |  |
| Not very supported | 3 (19) | 20 (19) | 23 (13) |  |
| Not at all supported | 0 (0) | 8 (8) | 10 (6) |  |
| **Body weight/overweight and obesity** | | | | 0.56 |
| Very supported | 3 (19) | 25 (24) | 40 (23) |  |
| Somewhat supported | 5 (31) | 22 (21) | 29 (17) |  |
| Neutral | 5 (31) | 27 (26) | 42 (24) |  |
| Not very supported | 2 (13) | 10 (10) | 19 (11) |  |
| Not at all supported | 0 (0) | 8 (8) | 8 (5) |  |
| **Stress/mental health** | | | | 0.21 |
| Very supported | 7 (44) | 36 (35) | 67 (39) |  |
| Somewhat supported | 2 (13) | 33 (32) | 43 (25) |  |
| Neutral | 5 (31) | 21 (20) | 24 (14) |  |
| Not very supported | 1 (6) | 8 (8) | 14 (8) |  |
| Not at all supported | 0 (0) | 4 (4) | 8 (5) |  |

CVD: cardiovascular disease. HDP: hypertensive disorders of pregnancy.

**Additional Table 6. Level of support women with a history of HDP perceived from general practitioners to make changes to lifestyle cardiovascular disease risk factors risk factors, by SES**

| **Subtype of HDP** | **Quintile 1 (n=26)** | **Quintile 2**  **(n=52)** | **Quintile 3 (n=89)** | **Quintile 4 (n=72)** | **Quintile 5 (n=54)** | **P-value** |
| --- | --- | --- | --- | --- | --- | --- |
| **CVD lifestyle risk markers*** | **Total n (%)** | | | | | |
| **Diet** | | | | | | 0.20 |
| Very supported | 9 (35) | 15 (29) | 16 (18) | 19 (26) | 13 (24) |  |
| Somewhat supported | 1 (4) | 10 (19) | 27 (30) | 19 (26) | 9 (17) |  |
| Neutral | 6 (23) | 13 (25) | 28 (32) | 16 (22) | 14 (26) |  |
| Not very supported | 6 (23) | 6 (12) | 5 (6) | 6 (11) | 9 (17) |  |
| Not at all supported | 1 (4) | 5 (10) | 2 (2) | 3 (4) | 3 (6) |  |
| **Physical activity** | | | | | | 0.40 |
| Very supported | 8 (31) | 14 (27) | 15 (17) | 16 (22) | 11 (21) |  |
| Somewhat supported | 2 (8) | 10 (19) | 26 (29) | 21 (29) | 10 (19) |  |
| Neutral | 6 (23) | 13 (25) | 26 (29) | 17 (24) | 15 (28) |  |
| Not very supported | 6 (23) | 6 (12) | 8 (9) | 10 (14) | 10 (19) |  |
| Not at all supported | 1 (4) | 5 (10) | 2 (2) | 1 (2) | 3 (6) |  |
| **Smoking status** | | | | | | 0.42 |
| Very supported | 5 (19) | 6 (12) | 8 (9) | 11 (16) | 5 (9) |  |
| Somewhat supported | 0 (0) | 4 (8) | 8 (9) | 4 (6) | 5 (9) |  |
| Neutral | 3 (12) | 3 (6) | 8 (9) | 8 (11) | 9 (17) |  |
| Not very supported | 2 (8) | 2 (4) | 0 (0) | 2 (3) | 2 (4) |  |
| Not at all supported | 1 (4) | 4 (8) | 2 (3) | 2 (3) | 0 (0) |  |
| **Alcohol consumption** | | | | | | 0.49 |
| Very supported | 6 (23) | 7 (14) | 10 (11) | 14 (19) | 6 (11) |  |
| Somewhat supported | 1 (4) | 5 (10) | 10 (11) | 8 (11) | 5 (9) |  |
| Neutral | 6 (23) | 4 (8) | 10 (11) | 11 (15) | 12 (22) |  |
| Not very supported | 2 (8) | 3 (6) | 2 (2) | 4 (6) | 3 (6) |  |
| Not at all supported | 1 (4) | 3 (6) | 2 (2) | 3 (4) | 0 (0) |  |
| **Sleep habits** | | | | | | 0.84 |
| Very supported | 7 (27) | 10 (19) | 13 (15) | 14 (19) | 9 (17) |  |
| Somewhat supported | 3 (12) | 8 (15) | 18 (20) | 18 (25) | 10 (19) |  |
| Neutral | 7 (27) | 14 (27) | 121 (24) | 17 (24) | 12 (22) |  |
| Not very supported | 6 (23) | 6 (12) | 14 (16) | 10 (14) | 10 (19) |  |
| Not at all supported | 1 (4) | 5 (102) | 3 (3) | 4 (6) | 5 (9) |  |
| **Body weight/overweight and obesity** | | | | | | 0.15 |
| Very supported | 10 (39) | 12 (23) | 20 (23) | 17 (24) | 19 (17) |  |
| Somewhat supported | 2 (8) | 10 (19) | 22 (25) | 15 (21) | 7 (13) |  |
| Neutral | 3 (12) | 13 (25) | 25 (28) | 18 (2) | 15 (28) |  |
| Not very supported | 5 (19) | 5 (10) | 3 (3) | 9 (13) | 9 (17) |  |
| Not at all supported | 3 (12) | 5 (10) | 2 (2) | 2 (3) | 4 (7) |  |
| **Stress/mental health** | | | | | | 0.35 |
| Very supported | 14 (54) | 20 (39) | 32 (36) | 28 (39) | 16 (30) |  |
| Somewhat supported | 4 (15) | 10 (19) | 31 (35) | 18 (25) | 15 (28) |  |
| Neutral | 4 (15) | 11 (21) | 11 (12) | 13 (18) | 11 (2) |  |
| Not very supported | 2 (8) | 2 (4) | 4 (5) | 8 (11) | 7 (13) |  |
| Not at all supported | 2 (8) | 4 (8) | 3 (3) | 1 (1) | 2 (4) |  |

CVD: cardiovascular disease. HDP: hypertensive disorders of pregnancy. SES: socioeconomic status. SEIFA: Socioeconomic Indexes for Areas are quintiles/codes that indicate socioeconomic status. 1=lowest socioeconomic areas and 5=highest socioeconomic areas.
